# Impact of Patient Personality on Adherence to Oral Anticancer Medications: An Opportunity?

**DOI:** 10.2196/57199

**Published:** 2024-10-30

**Authors:** Mahtab Jafari, Alex Shahverdian, Gelareh Sadigh, Richard A Van Etten

**Affiliations:** 1 Department of Pharmaceutical Scienes University of California, Irvine Irvine, CA United States; 2 Department of Radiological Sciences University of California, Irvine Orange, CA United States; 3 Department of Medicine University of California, Irvine Orange, CA United States

**Keywords:** cancer, medication adherence, medication persistence, Five-Factor Model, Type D personality, oncology, cancer medications, oral anticancer therapy, chemotherapy

## Abstract

Adherence to prescribed oral anticancer therapy is an important determinant of patient outcomes, including progression-free and overall survival. While many factors (eg, medication side effects and out-of-pocket costs, problems with insurance authorization, and timely medication refills) can affect adherence, one that is relatively unexplored is the impact of a patient’s attitude and personality. Patient personality influences medication adherence and persistence in nonmalignant chronic conditions such as cardiovascular disease and diabetes. In breast cancer and chronic myeloid leukemia, studies suggest that personality also affects adherence to oral chemotherapy which can be targeted to improve adherence. In this viewpoint, we highlight the opportunity of incorporating patient personality as interventions to oral cancer therapy adherence and discuss current barriers to implementation.

## Introduction

With acceleration in development of oral anticancer medications in recent years, a substantial number of patients with cancer are responsible for managing their medication. While oral anticancer medications have many advantages over parenteral chemotherapy, including eliminating the need for venous access devices, many patients struggle with adhering to their prescribed regimens. Whereas medication adherence rates among patients with chronic diseases on oral treatment are estimated at approximately 50%, adherence rates for oral anticancer medications are substantially lower, with studies reporting adherence rates as low as 30% to 46% in patients with cancer [1-3]. Similarly, persistence to oral anticancer medications, defined as continuing treatment for the prescribed duration of therapy, is also suboptimal; for example, at 12 and 24 months, treatment persistence in patients with gastrointestinal stroma tumors and chronic myeloid leukemia was reported to be 41% and 56%, respectively [4]. These are concerning statistics given that poor adherence to prescribed cancer therapy can lead to serious consequences such as disease progression, reduced treatment efficacy, increased symptom burden, an increased risk for recurrent cancer, and decreased overall survival [5-7].

Many patient-related factors can contribute to nonadherence and nonpersistence to prescribed therapies, including health literacy [8], social determinants of health including food insecurity and housing instability [9], out-of-pocket medication costs [10,11], patient age [12,13], number of prescribed medications [14], and medication side effects [15-17]. However, the impact of patient personality and psychosocial characteristics has remained relatively underexplored [18-22]. In this viewpoint, we review literature on the impact of personality on medication adherence and argue that developing patient education that is tailored toward each individual patient’s personality may improve anticancer medication adherence.

## Assessment of Personality Types

The psychological literature frequently assesses personality using the Five Factor Model (FFM) [23]. Also known as the universal model, the FFM is one of the most empirically supported personality models to date and consists of 5 personality categories (Table 1): openness, conscientiousness, extraversion, agreeableness, and neuroticism. An alternative model recognizes 4 personality types (types A, B, C, and D) [24]. The original categories of type A (competitive, ambitious) and type B (patient, creative) were first defined and studied in patients with cardiovascular disease [25], and were subsequently expanded to include types C (analytical, introverted) and D (anxious, negative). In particular, the type D personality is a trait associated with negative emotions such as worrying, and a lack of social interaction out of fear of disapproval (Textbox 1) [26-28]. The relationship between the FFM and ABCD personality models has not been fully defined, but type D subjects display FFM traits ranging from neurotic introversion with relatively low conscientiousness to stable extraversion with relatively high conscientiousness [29]. The Eysenck personality theory recognizes personality traits across 3 dimensions, extraversion/introversion, neuroticism/stability, and psychoticism/superego [30], and is commonly assessed using the Eysenck Personality Questionnaire-Revised Short Scale [31].

**Table 1:** The Five Factor personalities and associated adjectives (adapted from [23]).

Personality	Adjectives
Openness	Artistic, curious, imaginative, insightful, original, and wide interests
Conscientiousness	Efficient, organized, planful, reliable, responsible, and thorough
Extraversion	Active, assertive, energetic, enthusiastic, outgoing, and talkative
Agreeableness	Appreciative, forgiving, generous, kind, sympathetic, and trusting
Neuroticism	Anxious, self-pitying, tense, touchy, unstable, and worrying

**Characteristics of type D personality [26].**

**Type D traits**

- Tendency to experience negative emotions.
- Propensity to suppress the expression of emotions and behaviors in social contacts.
- Feeling of unhappiness, worry, irritability, and low self-esteem.
- Distance in social relations, introversion.

## Impact of Personality on Medication Adherence in Cardiovascular Disease and Diabetes

Association between patient personality assessed by the Five Factor Model and medication adherence has been studied in patients with cardiovascular disease [32]. In a recent study, patient personality was measured using the Japanese Ten-Item Personality Inventory for evaluation of the Big Five personality traits. A 12-item adherence scale measured medication compliance, health care provider collaboration, willingness to access medication information, and acceptance of needing to take medication. Having higher conscientiousness was significantly associated with greater medication compliance, patient-provider–shared decision-making, and willingness to access information about medications [32]. Conscientiousness has also been associated significantly with health-related quality of life, self-efficacy, and satisfaction with life in patients with cardiovascular disease [33]. The type D personality trait, a measure of low social interaction and negative affectivity, is frequently observed in patients with cardiovascular disease [34,35]. Type D individuals have significantly poorer medication adherence patterns in patients with myocardial infarction [36], heart failure [35,37], and acute coronary syndrome [38]. This is in addition to the type D personality being a significant predictor of mortality in patients suffering from coronary heart disease [34].

Another common disease where medication adherence and personality have been studied is diabetes. Low adherence is a known issue in diabetic patients, leading to increased adverse outcomes such as higher hemoglobin A_1c_ (HbA_1c_) levels and peripheral neuropathy [39]. More recently, studies have investigated the role the Five Factor personalities have in diabetes [40,41]. In one study, diabetics determined to possess the neuroticism trait based on the Eysenck Personality Questionnaire-Revised Short Scale were significantly less likely to be adherent to medication in bivariate analyses. The authors hypothesized an indirect relationship between adherence and neuroticism mediated through neuroticism’s association with a lack of social support and self-efficacy [40]. Another study also showed a significant negative relationship between neuroticism and adherence along with self-care behaviors [41], but found a significant positive relationship between agreeableness and adherence. Finally, conscientiousness has also been demonstrated to be significantly positively correlated to taking medications as prescribed in type 2 diabetics [42]. These trends are not exclusive to type 2 diabetes, as adolescents with type 1 diabetes who possessed the conscientiousness trait were significantly more adherent to insulin administration while those with the neuroticism trait showed a significantly negative correlation [43]. Like cardiovascular disease, type D personality has also been linked to poor medication adherence in type 2 diabetics [44,45] and to be associated with increased HbA_1c_ [45].

## Medication Adherence and Personality in Patients With Cancer

As in other chronic nonmalignant diseases, nonadherence and nonpersistence to oral anticancer medication can be associated with multiple patient-related factors, some of which may be specific to the type or stage of cancer diagnosis or the duration of the prescribed therapy. The mental impact that accompanies a diagnosis of cancer may trigger or exacerbate behaviors that tend to be associated with the specific personality type of a patient [46-48]. For example, it is possible that some patients with aspects of the type D personality may express negative social and affective traits when confronted with cancer. Relative to other chronic diseases like hypertension and diabetes, cancer therapy is unique in that patients are dealing with an imminent life-threatening condition with medications where the drug choices may be limited, and the side effects are substantial. Indeed, many studies in cancer patients identify medication side effects to be a major factor contributing to poor adherence and persistence [3,49-51]. It follows that a patient’s attitude and personality might have a major effect on coping with such symptoms. However, literature examining personality traits and adherence in patients with cancer is limited. A study that examined the link between the Five Factor Model and adherence to outpatient cancer therapies suggested that the 2 personality types of conscientiousness and agreeableness correlated with increased adherence [52], but the specific types of cancer and treatments were not explored in detail. Other studies have focused on aspects of a patient’s emotional state rather than on personality traits per se, as a functional relationship between personality type and the regulation of emotions has been documented [53-55]. For example, a review of psychosocial determinants of adherence to oral anticancer treatment found high levels of distress (anxiety and depression) to be a major factor contributing to nonadherence [56]. Medication beliefs have also been found to impact adherence to cancer medications [50,57-59].

Two cancer types where adherence has been studied in significant detail are early-stage breast cancer and chronic myeloid leukemia [7]. Patients with either of these vastly different malignancies share 2 characteristics: minimal symptoms arising from the cancer itself and a major impact of medication nonadherence on progression-free and overall survival. Patients with early-stage hormone-receptor positive breast cancer are frequently treated with oral medications targeting estrogen and progesterone signaling (adjuvant endocrine therapy [AET]) following surgical management of the primary tumor. Nonadherence and nonpersistence to prescribed AET have been shown in numerous studies to correlate with significantly reduced overall survival [60-62], particularly in Black women [63]. Side effects of AET represent a major factor associated with nonadherence in this population [49,64,65]. Patient personality has not been studied explicitly as a factor in AET adherence, but other studies have identified anticipatory positive emotions [66] and lower depressive symptoms associated with greater social support [67] to be associated with increased adherence.

Therapy of chronic myeloid leukemia (CML) has been revolutionized by Abelson 1 tyrosine kinase inhibitors (TKIs) such as imatinib (Gleevec). Most patients with CML achieve cytogenetic remission with TKI treatment [68,69] and enjoy age-adjusted normal life expectancy [70], but therapy must be lifelong for most patients [71]. Adherence and persistence to TKI therapy is of paramount importance to clinical outcomes of patients with CML, as missing just 1 dose a week is associated with suboptimal response [72] and treatment failure [73,74]. As a consequence, the factors associated with TKI adherence in CML have been studied extensively [75,76] and include out-of-pocket costs [77,78], long-term side effects [79,80], and dosing schedule [81]. In CML as in breast cancer, the impact of patient personality on medication adherence has been largely unexplored, but a recent study found that patients with either type A or type D personality (particularly negative affectivity) were more prone to TKI nonadherence [82].

## Can an Understanding of Patient Personality Be Leveraged to Improve Medication Adherence in Patients With Cancer?

A patient’s personality can inform differences in the way they think, behave and feel [83]. It can help predict their compliance with follow-up appointments, adherence to medications, and the tendency to accept and implement medical advice [84,85]. Furthermore, the personality of a patient likely influences other patient-related factors including emotional state, regulation, and stability [54,55]. The specific mechanisms through which a patient’s personality type might impact their adherence to medication have not been fully defined. It is possible that personality might have a direct effect on adherence, or a given personality trait might moderate the relationship between other factors that influence adherence, for example, between stress and levels of anxiety or depression. Previous studies of the moderating effects of personality on stress responses have yielded mixed results. For example, the neuroticism personality type has been found to moderate the relationship between stress and negative affect or health behaviors [86,87] and between medication beliefs and adherence during the COVID-19 pandemic [88], while another study demonstrated a correlation between personality and health trajectory but no moderating effect of personality type on the effect of stress on health outcomes [89]. Further studies are necessary to clarify the mechanistic relationship between personality and medication adherence and to inform strategies for intervention.

Given the current state of our knowledge, what opportunities exist to leverage a patient’s personality to improve their adherence to prescribed medication in general and in cancer specifically? One approach might be to attempt to alter or modify a patient’s personality toward one that is more favorable for medication adherence (for example, from type D to type A, or from neuroticism to conscientiousness). While there is general agreement that one’s personality can change, most adult personalities are relatively stable over time and the degree of any change is small [90,91]. Furthermore, attempts to change personality may be complicated by the challenges imposed by a cancer diagnosis. Although personality trait change has been recognized as a potentially fruitful area for health policy initiatives [92], there is a dearth of published research on this method to improve medication adherence. A better approach might be to adapt patient educational and motivational materials to an individual’s personality to ensure that the information is conveyed in a way that is most effective. Communications tailored to personality have been shown to be more effective than standard one size fits all messaging in advertising [93], education [94], and health care [95,96], but have not yet been applied to medication adherence.

For example, patients who exhibit a neurotic personality type could be more likely to experience negative emotions like irritability and anxiety following a cancer diagnosis, negatively impacting adherence [97]. A behavioral intervention strategy that acknowledges the neurotic patient’s emotions and uses positive psychology techniques could prove helpful in this case [98]. For a patient who is extroverted or outgoing, allowing a safe and nonjudgmental space to share their opinions before educating them on their medication usage could ensure improved listening and adherence. Since extroverts thrive on being creative, they could also be empowered to take control of their own health and identify strategies that help them remember to take medications. It is important to note, however, that each of the 5 personality traits in the FFM represent a range between 2 extremes [23]. For instance, the extraversion trait represents a continuum between extreme extraversion and extreme introversion. In general, however, since most people lie at neither end of the spectrum but somewhere in between, multiple strategies for each patient’s unique disposition would likely be more effective [23].

A more frequently applied strategy to increase medication adherence is to focus interventions on psychosocial factors, some of which may be associated with personality. Several recent studies in patients with breast cancer have used interventions focused on personal attitudes and values to increase adherence to AET [99,100]. Post hoc analysis of a randomized controlled trial found relaxation training to be more effective than cognitive behavioral therapy in improving adherence to AET [101]. A remotely delivered intervention based on personal values demonstrated feasibility and acceptability and showed promise in improving AET adherence [102]. In CML, an education program tailored to individual patients based on interviews and a designed set of distinct adherence aids improved TKI adherence in a randomized trial [103]. However, most efforts to improve TKI adherence have relied on analysis of large datasets to identify interventions and lack patient-focused approaches [104]. To address this, we (the authors) have launched a clinical trial aimed at better understanding the correlation between patient personality (the dominant trait as assessed by the FFM) and TKI adherence in patients with CML (ClinicalTrials.gov NCT06229860).

Before these strategies can be explored further in the real-world setting, existing FFM personality assessments currently used in cancer care or literature must be evaluated. Although assessments of patient personality often appear in medical records, they are usually 1-sided remarks limited to terms such as “pleasant,” “short-tempered,” or “difficult” and portray a rather superficial and incomplete perspective, which can in turn lead to biased intuitions [105,106], suboptimal care, and poor adherence. Instead, a structured and validated approach should be adopted to provide a more reliable breakdown of personality. A recent study examined the use of the 20-item Mini International Personality Item Pool (mini-IPIP) scale in adults with cancer and reported potential validity of the tool in oncologic clinical settings [107]. Despite being a shorter version compared with other full versions of FFM personality measures, such as the NEO-Five Factor Inventory [108], the mini-IPIP has also been widely cited in studies including healthy adults and illustrated sufficient internal reliability across diverse population samples [109]. Since the mini-IPIP is a 20-item questionnaire with potential internal and external validity, the tool could be reasonably administered to cancer patients. To facilitate smooth patient-provider interactions, patients could be requested to complete these assessments during a patient intake process or before an appointment through patient portals to allow providers ample time to review their personality profiles and prepare as needed before an encounter.

## Conclusion

Behavioral intervention studies that seek to address each personality type should be conducted to reinforce positive health behaviors and promote adherence. Instead of using a cookie-cutter approach to patient counseling, understanding each person’s unique personality, and adopting communication strategies that encourage optimal adherence can improve oncologic patient care. However, further research is needed to evaluate the impact of personality-specific medication counseling on adherence to oral anticancer medications. This includes validation studies that confirm the reliability of personality assessments in cancer patients, as well as studies that explore the effectiveness of psychological behavioral techniques on adherence in different personalities. At the same time, there are enough data to encourage research in this direction. We strongly believe that incorporating personality into oncological care will redefine how we approach patient care as a whole, especially in this age where personalized care models like precision medicine are on the rise.

## Abbreviations

AET: adjuvant endocrine therapy
CML: chronic myeloid leukemia
FFM: Five Factor Model
HbA1c: hemoglobin A1c
IPIP: International Personality Item Pool
TKI: tyrosine kinase inhibitors
